# Longer prostate stromal cell telomere length is associated with increased risk of death from other cancers

**DOI:** 10.3389/fmed.2024.1390769

**Published:** 2024-05-28

**Authors:** Joakin O. Mori, Elizabeth A. Platz, Jiayun Lu, Alexandria Brame, Misop Han, Corinne E. Joshu, Angelo M. De Marzo, Alan K. Meeker, Christopher M. Heaphy

**Affiliations:** ^1^Department of Medicine, Boston University Chobanian & Avedisian School of Medicine and Boston Medical Center, Boston, MA, United States; ^2^Department of Epidemiology, Johns Hopkins Bloomberg School of Public Health, Baltimore, MD, United States; ^3^Sidney Kimmel Comprehensive Cancer Center at Johns Hopkins, Baltimore, MD, United States; ^4^James Buchanan Brady Urological Institute, Johns Hopkins University School of Medicine, Baltimore, MD, United States; ^5^Department of Pathology, Johns Hopkins University School of Medicine, Baltimore, MD, United States; ^6^Department of Pathology and Laboratory, Boston University Chobanian & Avedisian School of Medicine, Boston, MA, United States

**Keywords:** cancer, mortality, prostate cancer, stromal cells, telomeres

## Abstract

**Background:**

Telomeres are located at chromosomal termini and function to maintain genomic integrity. Telomere dysfunction is a well-recognized contributor to aging and age-related diseases, such as prostate cancer. Since telomere length is highly heritable, we postulate that stromal cell telomere length in the tissue of a particular solid organ may generally reflect constitutive stromal cell telomere length in other solid organs throughout the body. Even with telomere loss occurring with each round of cell replication, in general, telomere length in prostate stromal cells in mid-life would still be correlated with the telomere length in stromal cells in other organs. Thus, we hypothesize that prostate stromal cell telomere length and/or telomere length variability is a potential indicator of the likelihood of developing future solid cancers, beyond prostate cancer, and especially lethal cancer.

**Methods:**

To explore this hypothesis, we conducted a cohort study analysis of 1,175 men who were surgically treated for prostate cancer and were followed for death, including from causes other than their prostate cancer.

**Results:**

In this cohort study with a median follow-up of 19 years, we observed that longer prostate stromal cell telomere length measured in tissue microarray spots containing prostate cancer was associated with an increased risk of death from other solid cancers. Variability in telomere length among these prostate stromal cells was possibly positively associated with risk of death from other solid cancers.

**Conclusion:**

Studying the link between stromal cell telomere length and cancer mortality may be important for guiding the development of cancer interception and prevention strategies.

## 1 Introduction

Dysregulation of telomeres, the protective caps of chromosome ends that are essential for maintaining genomic integrity (1), is a well-recognized contributor to aging and age-related diseases (e.g., prostate cancer) (2–4). We previously observed that the presence of shorter telomere lengths in prostate cancer-associated stromal cells is associated with a higher risk of metastasis and death from prostate cancer in men surgically treated for clinically localized prostate cancer even when accounting for currently used prognostic indicators (5, 6). Also, we found that shorter telomere lengths in prostate stromal cells was associated with increased odds of prostate cancer (7). For these studies, we focused on non-lymphocyte stromal cell populations (e.g., fibroblasts) because these cells play a vital role in the structural composition, organization, and integrity of the extracellular matrix (8). However, through telomere dysregulation (i.e., either shorter or longer lengths), these cells can become dysfunctional or senescent and exhibit the senescence-associated secretory phenotype (SASP). In the setting of emerging cancer, SASP could influence cancer progression to lethality (9, 10). As such, studying the link between stromal cell telomere length and cancer mortality is important for guiding the development of cancer interception and prevention strategies, as well as aiding in potential treatment decisions.

Since telomere length is highly heritable (11), we hypothesize that stromal cell telomere length in the tissue of a particular solid organ may generally reflect constitutive stromal cell telomere length in other solid organs throughout the body. Thus, telomere length in prostate stromal cells in mid-life would still be correlated with the telomere length in stromal cells in other organs, even accounting for telomere loss that occurs due to the end replication problem (12). Thus, we hypothesize that prostate stromal cell telomere length and/or telomere length variability among stromal cells is a potential indicator of the likelihood of developing future solid cancers, beyond prostate cancer, and especially lethal cancer.

Prior studies have investigated the link between telomere length and mortality, including from cancer, using genomic DNA isolated from peripheral blood leukocytes (13–17). However, the results have been mixed. For example, a UK Biobank cohort study using data from >450,000 people found that shorter baseline leukocyte telomere length was associated with increased overall mortality (14). Conversely, specifically for prostate cancer, one study found no association (15), while another observed longer (16) telomeres in circulating leukocytes appear to be associated with prostate cancer mortality. Leukocyte telomere lengths vary due to differences in cell lineage and response to physiological processes; thus, these measurements are dynamic and can be unreliable as a reflection of constitutive telomere lengths for an individual. Leukocytes develop from hematopoietic stem cells present in the bone marrow and display telomerase activity during certain stages of development (18). Moreover, telomerase activity has been detected in circulating leukocytes, and factors such as systemic infection and other health factors (e.g., obesity), lifestyle factors (e.g., physical inactivity), and environmental exposures (e.g., smoking) may affect telomerase levels and telomere lengths (19–22). However, it is unlikely that the factors linked to dynamic changes in leukocyte telomere length would affect telomere length in all cells, including stromal cells, equally throughout the body.

Thus, to explore the hypothesis that stromal cell telomere length in one organ (prostate) is associated with future risk of death from solid cancers, we conducted a cohort study analysis of 1,175 men who were surgically treated for prostate cancer and were followed for death, including from causes other than their prostate cancer. In this study, we observed that longer prostate stromal cell telomere length measured in tissue microarray (TMA) spots containing prostate cancer was associated with an increased risk of death from other solid cancers.

## 2 Materials and methods

### 2.1 Study populations and designs

We used data on prostate stromal cell telomere measured in TMAs containing prostate cancer that we previously generated for five cohorts of men surgically treated for clinically localized prostate cancer: Recurrence Nested Case-Control Study (5), Intermediate-High-Risk Case-Cohort I and II studies (5), the Race Disparity Cohort from the Prostate Cancer Biorepository Network (PCBN) (23), and a cross-sectional cohort designed specifically to evaluate the association of obesity and weight change with prostate tissue biomarkers (Obesity Cohort) (24). The study was approved by the Institutional Review Board at the Johns Hopkins University School of Medicine (Baltimore, MD).

The demographic and pathological characteristics of these cohorts have been described in previous studies (5, 23, 24). Briefly, all cohorts comprised men treated at the Johns Hopkins Hospital and who were followed as part of a research protocol. The Recurrence Nested Case-Control Study included 1,048 men (524 cases, 524 controls) without hormonal or radiation therapy before surgery or adjuvant therapy before recurrence. The cases were men with biochemical recurrence, metastasis, or prostate cancer death after surgery by 2004, whichever came first. The controls were men who had not recurred by the date of the matched case. Cases and controls were matched by age, race, pathological stage, and Gleason sum. The Intermediate-High-Risk Case Cohorts I and II included men with intermediate or high-risk prostate cancer. Risk assessment for Cohort I was based on the Cancer of the Prostate Risk Assessment Post-surgical (CAPRA-S) score, while that of Cohort II was based on the D'Amico classification at the time of biopsy. In both cohorts, men with metastatic disease or positive lymph nodes detected by imaging before surgery were excluded. The subcohort for Intermediate-High-Risk Case Cohort I included 416 men and 80 men outside of the subcohort who progressed. The subcohort for Intermediate-High-Risk Case Cohort II included 235 men and 121 men outside of the subcohort who progressed. The Race Disparity Cohort included self-identified Black and White men enriched in either high or low-grade disease (23). The high-grade group included 57 Black and 59 White men treated between 2014 and 2016 and frequency matched on pathologic grade, age at diagnosis, Gleason sum, and pathologic stage. The low-grade group included 77 Black and 75 White men treated between 2000 and 2010, and frequency matched on age, prostatectomy date, surgeon, Gleason sum, stage, and margin status. Finally, the Obesity Cohort is a cross-sectional study designed to evaluate the association of obesity and weight change with prostate tissue biomarkers. Men were sampled from a retrospective cohort study of 1,337 men with clinically localized prostate cancer surgically treated between 1993 and 2006 who returned a lifestyle questionnaire. A total of 291 men were jointly categorized by weight change from 5 years before prostatectomy to 1 year after and BMI at 1 year after prostatectomy and frequency matched by age, race, and pathologic stage and grade.

For this analysis, we formed an analytic cohort from the controls from the Recurrence Nested Case-Control Study (the first time they were sampled, if sampled more than once), the subcohorts from the Intermediate-High-Risk Case-Cohorts I and II, and all men from the Race Disparity Cohort and Obesity Cohort. Among the five cohorts, 47 men were included in more than one cohort; we randomly selected one observation for the analytic cohort. Overall, the analytic cohort included 1,183 men. The years of prostatectomy spanned 1992 to 2016; eight men were excluded because year was not available. Follow-up of each man began at the date of prostatectomy and ended on 11/07/2022. The final analytic cohort included 1,175 men who underwent a prostatectomy for clinically localized prostate cancer, among whom 208 deaths were ascertained over a median follow up of 19.0 years.

### 2.2 Measurement of stromal cell telomere length

Formalin-fixed paraffin-embedded (FFPE) tissue samples from the 5 cohorts were arrayed on TMAs and we used a semi-automated, optimized method to measure telomere length that we previously described (5). Briefly, this semi-automated method is based on performing telomere-specific fluorescence *in situ* hybridization (FISH) combined with multiplex immunofluorescence to detect a basal cell-specific cytokeratin, prostate epithelial-cell specific nuclear markers (NKX3.1 and FOXA1), and lymphocyte-specific markers (CD3 and CD20) using FFPE TMA slides. This staining process is then followed by semi-automated slide scanning and multi-channel acquisition of fluorescent microscopy images using the TissueFAXS Plus (Tissue Gnostics, Vienna, Austria) automated microscopy workstation, which contains an eight-slide ultra-precise motorized stage and utilizes a Zeiss Z2 Axioimager microscope (Zeiss, Oberkocken, Germany). Cell-type specific telomere and nuclear DNA content data are then obtained from these collected images via semi-automated image analysis allowing us to measure telomere length in all cells of the specified type in focal plane without selection by the operator. For each man and for each cell, the ratio of Cy3 dot sum intensity (telomere signal) and DAPI area sum intensity (nuclear signal) was calculated and multiplied by 1,000; this ratio represents the telomere ratio. The telomere ratio for each nucleus compensates for differences in nuclear cutting planes and ploidy. For each man, we determined the median and standard deviation telomere length among his assessed stromal cells.

### 2.3 Ascertainment of cause of death

Men surgically treated for prostate cancer at Johns Hopkins and followed under a research protocol are routinely linked with the National Death Index as the primary method to ascertain fact, cause, and date of death. Reports from next of kin are also used. For this analysis, we linked the men in the five cohorts with the death information, and classified underlying causes of death as from prostate cancer, solid cancers other than prostate (documented primary disease and specific cancer type), and non-cancer causes of death (inclusive of non-disease reasons such as accidents).

### 2.4 Statistical analysis

Based on our prior work (5, 6), we defined shorter prostate stromal cell telomere length as below the 66th percentile of the distribution of median telomere length. We defined more variable stromal cell telomere length as above the 66th percentile of the distribution of the standard deviation of telomere length. We also divided the distribution of stromal cell telomere length and variability in telomere length into deciles. We defined all cutpoints separately by cohort because we measured stromal cell telomere length in separate batches over time.

We used Cox proportional hazards regression to estimate the hazard ratio (HR) and 95% confidence interval (95% CI) of all-cause mortality, death from solid cancers other than prostate, and death from other causes associated with shorter or more variable stromal cell telomere length. For the outcome death from solid cancers other than prostate, we censored men who died of prostate cancer or of other non-cancer causes at their date of death. Likewise, for the outcome death from other causes, we censored men who died of prostate cancer or solid cancers other than prostate at their date of death. In the model, we adjusted for age (continuous), race (Black, White, Other), and calendar year of prostatectomy (continuous). To test for trend, we entered into the model an ordinal variable for deciles of telomere length or variability in telomere length. For all analyses, we used SAS v. 9.4 (Cary, NC, USA).

## 3 Results

### 3.1 Characteristics of men in the analytic cohort

In the analytic cohort of 1,175 men, mean age at prostatectomy was 58.2 years, 81% were White, and 29.9% had a Gleason sum of 7 (4 + 3) or higher. Table 1 shows the demographic and pathologic characteristics of the analytic cohort by shorter (*N* = 783) vs. longer (*N* = 392) telomere length, or more variable (*N* = 392) vs. less variable (*N* = 783) telomere length in prostate cancer-associated stromal cells. Mean age slightly differed between the shorter and longer telomere length groups (58.6 vs. 57.6; *p* = 0.02). Mean age did not differ between the more and less variable telomere length groups (57.8 vs. 58.5 years; *p* = 0.1). Gleason sum did not differ between men with shorter and longer stromal cell telomeres or between men with more and less variable stromal cell telomeres. Overall, the median follow-up time was 19 years and did not significantly differ between the different telomere groups.

**Table 1:** Telomere length and telomere length variability in prostate cancer-associated stromal cells, 1,175 men who underwent prostatectomy for clinically localized prostate cancer at Johns Hopkins Hospital.

	**Overall**	**Telomere length**		***p*-value**	**Telomere length variability**		***p*-value**
		**Shorter** ^*^	**Longer** ^*^		**More** ^**^	**Less** ^**^	
**Number of men**							
	1,175	783	392		392	783	
**Mean age (years)**							
	58.2	58.6	57.6	0.02	57.8	58.5	0.1
**White (%)**							
	81.0	81.1	80.9	0.6	77.8	82.6	0.1
**Prostatectomy Gleason sum (%)**							
≤6	32.7	33.7	30.6	0.1	31.9	33.1	0.1
3 + 4	37.0	34.7	41.6		41.1	35.0	
4 + 3	12.9	14.3	10.2		10.7	14.1	
8–9	17.0	17.1	16.8		15.6	17.8	
**Median follow up time (years)**							
	19.0	19.0	20.0	0.2	20.0	19.0	0.1

* Shorter (bottom and middle tertiles) vs. Longer (top tertile).

** More variable (top tertile) vs. less variable (middle and bottom tertiles).

### 3.2 Distributions of death by stromal cell telomere length and telomere length variability

In the shorter and longer stromal cell telomere length groups, 18.5% (*N* = 145) and 16.1% (*N* = 63), respectively, of the men were deceased. In the more and less variable telomere length groups, 16.3% (*N* = 64) and 18.4% (*N* = 144), respectively, of the men were deceased. The proportions of death from prostate cancer, solid cancers (lung or bronchus, colon, esophagus, kidney, pancreas, and stomach cancer) other than prostate cancer, other cancers, and causes of death other than cancer were similar across the telomere length and variability groups (Table 2). Death from causes other than cancer was the most common, followed by death from prostate cancer, death from other cancers, and death from solid cancers other than prostate cancer.

**Table 2:** Distribution of deaths by telomere length and variability in telomere length among prostate cancer-associated stromal cells, 1,175 men who underwent prostatectomy for clinically localized prostate cancer at Johns Hopkins Hospital.

	**Telomere length**		**Variability in telomere length**	
	**Shorter** ^*^	**Longer** ^*^	**More** ^**^	**Less** ^**^
Total Number of men	783	392	392	783
Death from all causes (%)	145 (18.5)	63 (16.1)	64 (16.3)	144 (18.4)
All death except prostate cancer (%)	98 (12.5)	44 (11.2)	47 (12.0)	95 (12.1)
All non-cancer death (%)	73 (9.3)	31 (7.9)	30 (7.7)	74 (9.5)
All cancer death except prostate cancer (%)	25 (3.2)	13 (3.3)	17 (4.3)	21 (2.7)
Solid cancer death except prostate cancer (%)^***^	5 (0.6)	8 (2.0)	7 (1.8)	6 (0.8)

* Shorter = bottom and middle tertile vs. Longer = top tertile.

** More variable = top tertile vs. less variable = middle and bottom tertiles.

*** Solid cancer included lung or bronchus (N = 5), colon (N = 1), esophagus (N = 1), kidney (N = 1), pancreas (N = 2), and stomach cancer (N =3).

### 3.3 Telomere length and variability and risk of death from solid cancers other than prostate cancer

Compared to shorter telomere length, longer telomere length was significantly associated with an increased risk of death from other solid cancers (HR = 3.30, *p* = 0.03; Table 3). Compared with less variable telomere length, more variable telomere length appeared to be associated with an increased risk of death from other solid cancers (HR = 2.35), although the association was not statistically significant (*p* = 0.13).

**Table 3:** Association of telomere length and variability in telomere length among prostate cancer-associated stromal cells with death from causes other than prostate cancer, 1,175 men who underwent prostatectomy for clinically localized prostate cancer at Johns Hopkins Hospital.

	**Cases/person years**	HR^****^	**95% CI**	***p*-value**	*p*-trend^*****^
**All death except prostate cancer**					
Shorter	98/14,085	1.00	Ref		0.5
Longer^*^	44/7,241	0.98	0.69–1.41	0.9	
Less variable^**^	95/14,041	1.00	Ref		0.8
More variable	47/7,285	0.99	0.70–1.41	1.0	
**All non-cancer death**					
Shorter	73/14,085	1.00	Ref		0.8
Longer^*^	31/7,241	0.94	0.62–1.43	0.8	
Less variable^**^	74/14,041	1.00	Ref		0.3
More variable	30/7,285	0.80	0.52–1.23	0.3	
**All cancer death except prostate cancer**					
Shorter	25/14,085	1.00	Ref		0.9
Longer^*^	13/7,241	1.12	0.57–2.20	0.7	
Less variable^**^	21/14,041	1.00	Ref		0.3
More variable	17/7,285	1.68	0.88–3.19	0.1	
**Solid cancer death except prostate cancer** ^****^					
Shorter	5/14,085	1.00	Ref		0.1
Longer^*^	8/7,241	3.30	1.09–10.31	0.03	
Less variable^**^	6/14,041	1.00	Ref		0.02
More variable	7/7,285	2.35	0.79–7.03	0.1	

* Shorter = bottom and middle tertiles vs. longer = top tertile.

** More variable = top tertile vs. less variable = middle and bottom tertiles.

*** Solid cancer included lung or bronchus (N = 5), colon (N = 1), esophagus (N = 1), kidney (N = 1), pancreas (N = 2), and stomach cancer (N = 3). Of note, solid cancer death was also counted in other cancer death.

**** Hazard ratios estimated from Cox proportional hazards regressions models with the adjustment for age, race, and year of prostatectomy.

***** *p*-trend for deciles.

## 4 Discussion

In this cohort study with a median follow-up of 19 years, we observed that longer telomere length in prostate cancer-associated stromal cells in men surgically treated for prostate cancer is positively associated with an increased risk of death from other solid cancers. Variability in telomere length among prostate cancer-associated stromal cells was possibly positively associated with risk of death from other solid cancers. To our knowledge, no previous study has investigated prostatic stromal cell telomere length measured in individual cells in fixed tissues and risk of death from solid cancers.

Resident cells in the adult prostatic stromal component are predominantly terminally differentiated cells including fibroblast and smooth muscle cells (9, 25). These cells have limited ability to dynamically alter their telomere length, but we previously showed that smoking was associated with telomere length in stromal cells (26). Importantly, the effect of smoking on stromal cell telomere length variability was observable up to 10 years after smoking, suggesting that telomere alterations in stromal cells are gradual and relatively stable. This makes stromal cell telomere length and length variability a more reliable prognostic tool compared to the commonly studied circulating leukocyte telomere length and length variability.

Telomere length in peripheral blood leukocytes has been studied extensively as a biomarker of human aging and disease. Shorter telomere length in leukocytes is associated with increased risk of several aging-related diseases, as well as overall mortality and decreased human life span (17). Conversely, longer leukocyte telomere length may be associated with increased risk for certain cancer types (27, 28). However, leukocyte telomere lengths may not represent the most relevant measurement, as it is influenced by inherited genetic variation, and can be easily modified by lifestyle and environmental factors.

Our study has a number of strengths. This is a large cohort analysis consisting of 5 different study designs. In contrast to the commonly used measurement of mean telomere length from bulk peripheral blood leukocytes, assumed to be representative of telomere lengths throughout the body, our methodology allows us to examine telomere length at the level of individual cells in specific histological compartments within a tissue of interest; here, the prostatic stroma. Additionally, we were able to link with the National Death Index as the primary method to ascertain fact, cause, and date of death. However, other aspects of the work warrant discussion. The method we developed to determine the telomere status of prostate stromal cells does not directly determine actual telomere length, although the relative length is estimated and is linearly related to telomere length. Furthermore, although we were able to exclude stromal lymphocytes from our telomere length measurements, the precise identity of the remaining stromal cells remains unknown; thus, it is possible that further technical refinements to allow more precise cell identification may lead to improved associations between telomere length measurements and risk of cancer mortality and/or provide clues to the biological mechanism underlying these associations. In this current study, we analyzed prostate cancer stromal cell telomere length measurements to test the underlying rationale and hypothesis. However, while we postulate that these observed associations may also be true for stromal cell populations in other (cancerous or non-cancerous) tissues, further studies would be warranted. Finally, while this is a large study with long follow-up, this study represents the analysis from a cohort comprised of all males who were surgically treated for prostate cancer from a single institution and a relatively low number of events (i.e. deaths from solid tumors other than prostate).

In conclusion, in a cohort of 1,175 men, we observed that longer prostate cancer-associated stromal cell telomere length was associated with an increased risk of death from other solid cancers. Further study of the mechanistic link between telomere biology and cancer mortality may help guide the development of future cancer interception and prevention strategies.

## Data availability statement

The datasets presented in this article are not readily available, because the data will be shared upon review and completion of appropriate IRB and data use agreements. Requests to access the datasets should be directed at: CH; heaphyc@bu.edu or AM; ameeker1@jhmi.edu.

## Ethics statement

The studies involving humans were approved by Institutional Review Board at the Johns Hopkins University School of Medicine (Baltimore, MD). The studies were conducted in accordance with the local legislation and institutional requirements. The participants provided their written informed consent to participate in this study.

## Author contributions

JM: Conceptualization, Writing—original draft, Writing—review & editing. EP: Conceptualization, Writing—original draft, Writing—review & editing, Formal analysis, Supervision. JL: Formal analysis, Writing—review & editing. AB: Writing—review & editing, Data curation. MH: Data curation, Writing—review & editing, Supervision. CJ: Data curation, Writing—review & editing. AD: Writing—review & editing, Conceptualization, Supervision. AM: Conceptualization, Supervision, Writing—review & editing, Writing—original draft. CH: Conceptualization, Supervision, Writing—original draft, Writing—review & editing.
